# Experimental system of care coordination for the home return of patients with metastatic cancer: a survey of general practitioners

**DOI:** 10.1186/s12875-022-01891-9

**Published:** 2022-11-17

**Authors:** Laëtitia Gimenez, Vladimir Druel, Anastasia Bonnet, Cyrille Delpierre, Pascale Grosclaude, Marie-Eve Rouge-Bugat

**Affiliations:** 1 Département Universitaire de Médecine Générale – Université Toulouse III Paul Sabatier, 133 Route de Narbonne, 31062 Toulouse Cedex, France; 2 Faculté de médecine, CERPOP - UMR 1295 INSERM - Université Toulouse III Paul Sabatier, 37 allées Jules Guesde -, 31000 Toulouse, France; 3 Maison de Santé Pluriprofessionnelle Universitaire La Providence, 1 avenue Louis Blériot –, 31500 Toulouse, France; 4 Institut Universitaire du Cancer Toulouse – Oncopole, 1, avenue Irène Joliot-Curie –, 31059 Toulouse Cedex 9, France

**Keywords:** Care coordination, Cancer, General practitioner, Satisfaction

## Abstract

**Background:**

To promote improved coordination between general practice and hospital, the French clinical trial CREDO (“Concertation de REtour à DOmicile”) is testing an innovative experimental consultation for patients with metastatic cancer who are returning home. This consultation involves the patient, the patient’s referring GP (GP_ref_) and a GP with specific skills in oncology (GP_onc_) in a specialized care center. The objective of our study is to explore the satisfaction of GPs_ref_ about this consultation, in the phase of interaction between GP_onc_ and GP_ref_.

**Methods:**

This observational, cross-sectional, multicenter study explored the satisfaction of GPs_ref_ who had participated in this type of consultation, via a telephone survey.

**Results:**

One Hundred GPs_ref_ responded to the questionnaire between April and September 2019 (overall response rate: 55%). 84.5% were satisfied with the consultation, and the majority were satisfied with its methods. Half of the GPs_ref_ learned new information during the consultation, three-quarters noted an impact on their practice, and 94.4% thought that this type of coordination between the GP_ref_ and the oncology specialist could improve general practice - hospital coordination.

**Conclusions:**

For GPs, the CREDO consultation seems to be practical and effective in improving the coordination between general medicine and hospital. GPs would benefit from such coordination for all patients with cancer, several times during follow-up and at each occurrence of a medically significant event.

**Supplementary Information:**

The online version contains supplementary material available at 10.1186/s12875-022-01891-9.

## Background

The general practitioner (GP) has a central role in the different phases of cancer patient management [1–4], including in early treatment phases [5, 6]. However, exchanges between general practice and hospital remain fragmented [7]. In the active phase of anti-neoplastic treatment, patients first seek the advice of their oncologist in the event of an adverse effect or complication [8–10]. When patients return home, lack of medical information may present a source of difficulties for the patient’s referring GP.

Several initiatives have emerged in recent years to develop tools for effective care coordination between hospital and general practice for patients with cancer [11]. Implementation methods are diverse: standardized mailing, use of electronic support and relational exchanges between health professionals. The timing of information exchange between health professionals can also vary: during the initial oncology consultation, during the multidisciplinary consultation [12], during the announcement of the diagnosis of cancer [13], for shared decision-making of cancer treatment decisions [6], or at the time of discharge from hospital [14]. The quality of information transmission impacts the quality of care, the satisfaction of patients and GPs, and results in more frequent use of primary care, and a decrease in the number of hospitalizations due to adverse effects of anti-neoplastic treatments [15]. In addition, the involvement of GPs in the design and effective implementation of the tools seems to be one of the keys to effective care coordination between primary and hospital care [16].

Patients with metastatic cancer are not numerous in the active files of GPs but they are at risk of developing potentially serious complications or side effects. A clear and rapid communication of appropriate information is therefore essential for the smooth running of care, as well as for quality of life and for keeping patients at home in good conditions. Currently, in the French health care system, when a patient with cancer presents complications from his/her pathology or side effects due to his/her anti-neoplastic treatment, several solutions are possible depending on the severity, the day of the week, the time of day and the area where he/she lives:

1. Either the referring GP can receive the patient in consultation at his/her private practice or visit him/her at home. If necessary, he/she can obtain the advice of the referring oncologist from the specialised care centre by telephone to help him/her with the care management.
2. Or the patient goes to a local hospital which does not necessarily have an oncology department. In this case, the emergency or inpatient department can contact the GP and/or the referring oncologist at the specialised care centre by telephone to seek their advice on care management.
3. Or the patient goes directly to the specialised oncology care centre, especially if the situation is serious and if he/she lives close to the centre. In this case, communication with the referring oncologist is direct.

In most cases, a paper liaison file containing the care plan, the treatment protocol and the contacts of the referring professionals is given to the patient so that he/she can have it with him/her wherever care is provided. The limitations of this organisation may be multiple: the paper liaison file may be forgotten by the patient; the referring GP or the local hospital may lack prior information or competence to manage the patient; the referring GP and/or oncologist may be unavailable to answer telephone calls when complications/side effects occur.

In order to better involve GPs in these exchanges and the management of patients with cancer in the active treatment phase, a French team has been experimenting with an innovative general practice-hospital coordination model. The ongoing CREDO (“Concertation de REtour à DOmicile”) clinical trial is evaluating the efficacy of an organized “return home” consultation compared to the standard of care for patients with metastatic cancer. We took advantage of this multicenter, randomized the multi-center, randomized, open-label, prospective trial (described in Methods) that had already identified and involved the referring general practitioners (GP_ref_) of patients included in the trial, to implement an ancillary study focused on the satisfaction of these GPs. The primary objective of this ancillary study was to explore the satisfaction of GPs_ref_ participating in the CREDO trial about the exchanges occurring during this experimental system of care coordination. The secondary objectives were to gather the opinion of these GPs about the modalities and the contribution of CREDO exchanges for the care of their patients with metastatic cancer.

## Methods

### Overview

This study was conceived as an ancillary study of the multi-center, randomized, open-label, prospective CREDO trial. We took advantage of this trial to examine the satisfaction of GPs_ref_ participating to CREDO about the exchanges occurring during this experimental system of care coordination, through a cross-sectional survey.

### Description of the CREDO experimental system of care coordination

**Fig. 1:**
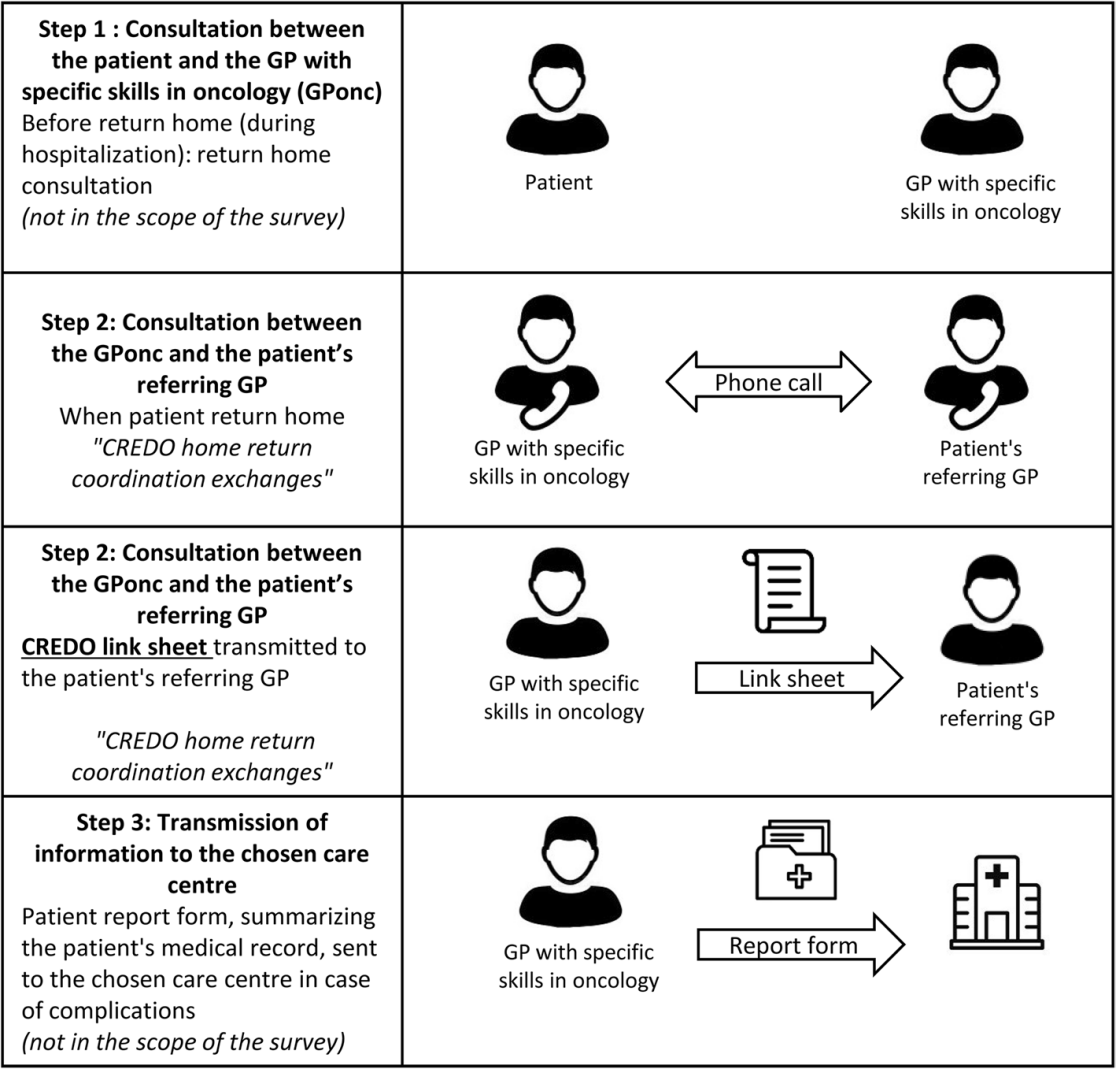
Representation of the CREDO standard and the steps included or not in the scope of the survey

The CREDO clinical trial has been registered with the ClinicalTrials.gov identifier: NCT02857400 on 05/08/2016. It has been running since July 2017 in two specialized cancer care centers in southern France (Occitania region). Patient inclusion criteria are: be over 18 years old; be treated with a first cycle of metastatic chemotherapy in a specialized cancer care center; have a metastatic solid cancer, regardless of the organ; and be returning home after treatment administration. Patients are randomized in two arms: standard arm (conventional management) or intervention arm (CREDO management). In the standard arm, representing the current standard of care, discharge information documents are sent by e-mail or fax to the GP_ref_ on the day of the patient’s discharge. In the intervention arm, a “return home” consultation is carried out in three steps between the different actors (Fig. 1):

1. First step: Consultation between the patient and the GP_onc_. A return home consultation is carried out during hospitalization between the patient and a GP (GP_onc_) with specific skills in oncology integrated in the specialized cancer care centre (including data collection on patient’s socio-demographic and medical information and patient’s management choices).GP_onc_ are already practising in some specialised care centres in France. GP_onc_ are GP who have completed a year of additional training in oncology services in order to be able to get more specifically involved in the care of patients with cancer.
2. Second step: Consultation between the GP_onc_ and the patient’s GP_ref_. A “CREDO link form” summarizing a telephone consultation between the GP_onc_ and GP_ref_ is transmitted to the GP_ref_ on the day of the patient’s discharge (transmission of information about patient’s state of health and current treatment opinion of GP_ref_ about the place of care desired by the patient in case of complications). An information sheet on the side effects of the anti-neoplastic treatment is also sent to the GP_ref_;
3. Third step: Transmission of information to the local care centre. A patient report form, summarizing the patient’s medical record, is transmitted to the chosen care structure in the event of a complication, when it is different from the specialized cancer care centre. This report form is sent as soon as the patient returns home.

The main outcome of the trial is the number of unscheduled visits of the patient to the care centers, after the first cycle of metastatic chemotherapy: consultations and hospitalizations in specialised or non-specialised cancer care centers.

All the patients are followed for 1 year.

### Study design

Our study focuses on the satisfaction of GPs_ref_ involved in the second part of the CREDO experimental system, represented by the transfer of information between GP_onc_ and GP_ref_. This phase is called “CREDO home return coordination exchanges” or “CREDO exchanges” in our study. We evaluated the satisfaction of GPs_ref_ with this system via telephone interviews, as part of a multi-center, cross-sectional study.

### Study population

The population is represented by the GPs_ref_ of the patients with metastatic cancer included in the experimental arm of the CREDO trial between July 2017 and February 2019.

### Development of the questionnaire

The questionnaire was developed on the basis of a literature review and validated by a group of 5 researchers including 4 GPs (LG, VD, AB and MERB) and an epidemiologist (PG).

The questionnaire consisted of two parts (Additional file 1). The first part (15 questions) focused on the satisfaction of the GPs_ref_. The first 10 questions asked GPs_ref_ about their experiences with CREDO exchanges. The last 5 questions could be completed even if the GP_ref_ interviewed could not remember the CREDO exchanges. The questionnaire was submitted to the GP_ref_ preferably by telephone, but could also be returned either by email or post mail. In case the GP_ref_ was unreachable after three call attempts, an e-mail or post mail was sent to the GP_ref_, followed by a fourth telephone call. In case of refusal of the telephone call, the questionnaire was sent by e-mail or post.

### Outcomes

The primary outcome was the satisfaction of the GPs_ref_ with the CREDO home return coordination exchanges, defined as a binary variable (“satisfied”/“rather satisfied” versus “rather unsatisfied”/“unsatisfied”). Secondary outcomes were the responses to all other binary or multi-choice questions.

### Analysis

Socio-demographic characteristics of the participating GPs_ref_, as well as the variables of interest, particularly GP_ref_ satisfaction, were described with numbers and percentages for the qualitative variables, and mean and standard deviations for the quantitative variables. In order to compare the nominal qualitative variables between the “satisfied” and “dissatisfied” groups in the study, a Fisher’s exact test was used as at least one of the expected values was less than five. For the comparison of the quantitative variables, the non-parametric Wilcoxon-Mann-Whitney test was used, as variances were homogeneous but non-normally distributed. The alpha significance level used for these tests was 0.05.

In the last open-ended question, GPs_ref_ could propose their own ideas for improving the general practice-hospital link. The key ideas for improving this coordination were extracted from their spontaneous verbatim and classified into seven main categories: type of information to be better shared, type of interlocutor, ideal characteristics of the interlocutor, type of communication medium, ideal characteristics of the communication medium, ideal characteristics of the communication and points to be improved in hospital practice.

## Results

### Characteristics of the population

**Fig. 2:**
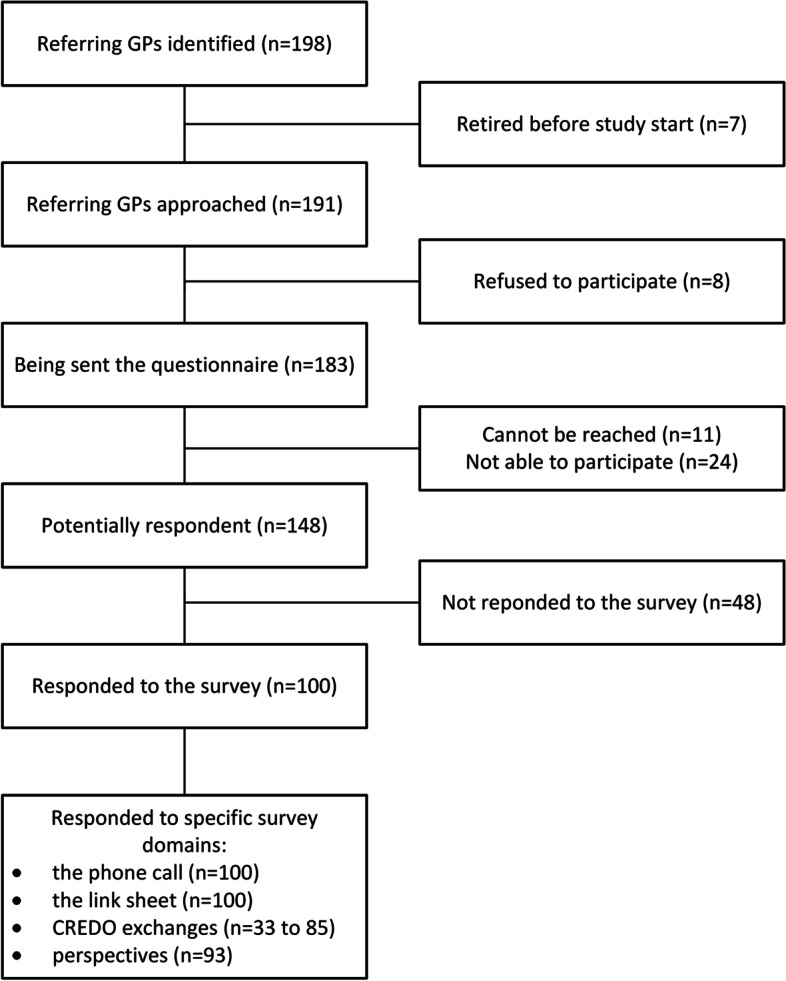
Participant flow diagram

We identified 198 GPs_ref_ concerned by the experimental arm of the patients included in the CREDO trial between July 21 2017 and February 20 2019. We conducted calls, emails and mailings between April 10, 2019 and September 27, 2019. After excluding GPs who retired before the start of the study, 183 GP_ref_ were approached, and 100 GP_ref_ finally responded to the questionnaire, as detailed in the study flow chart represented in Fig. 2.

The overall response rate was 55%, with 60% of the responses provided by phone (average time for completion, 14 minutes), 32% by email and 8 by mail. The response rate varied from 33 to 100% according to the questions considered. The characteristics of the GPs_ref_ sample are presented in Table 1.

**Table 1:** Characteristics of study participants and their satisfaction with CREDO exchanges

	Satisfied (***n*** = 49)	Not satisfied (***n*** = 9)	***p***-value	All respondents (***n*** = 100)^a^
**Gender**			0.467^1^	
Female	19 (79.2)	5 (20.8)		33
Male	30 (88.2)	4 (11.8)		67
**Mean age (SD)**	48.1 (11.6)	43.7 (11.5)	0.273^2^	49.9 (11.2)
**Location of practice**			0.999^1^	
Urban	18 (85.7)	3 (14.3)		37
Semi-rural	19 (82.6)	4 (17.4)		35
Rural	12 (85.7)	2 (14.3)		28
**Type of practice**			0.157^1^	
Sole practitioner	13 (92.9)	1 (7.1)		30
Group practice of several GPs	25 (89.3)	3 (10.7)		42
Group practice of several medical or paramedical specialties	5 (62.5)	3 (37.5)		15
Multidisciplinary Healthcare Center	6 (75)	2 (25)		13
**University lecturer**	13 (81.3)	3 (18.8)	0.696^1^	28
**Mean duration of practice (in years (SD)**	16.2 (12.7)	12.6 (11.3)	0.458^2^	18.6 (12.4)
**Degree or additional training in oncology**	4 (77.8)	2 (22.2)	0.231^1^	12

^1^ Fisher’s exact test ^2^ Wilcoxon-Mann-Whitney test

^a^Of the 100 respondents, 58 GPs answered the questions about satisfaction with CREDO exchanges

### Information about the phone call

As detailed in Table 2, less than half of the GPs_ref_ (40%) recalled being contacted by telephone to exchange information about their patient. The timing of the call was considered appropriate by 95% of the respondents. The duration of the call was unanimously described as appropriate, and 90% of respondents considered the choice of telephone calls to be appropriate for this type of exchange. The fact that the caller was a physician facilitated the exchanges for 92% of the respondents.

**Table 2:** Details of the responses to the questionnaire with number and percentage in agreement with the question

	N (%)
**Responded to the survey in general (*n*** **= 100)**	
**Concerning the phone call (*n*** **= 100)**	
Remembered receiving a call to exchange information on the patient’s state of health and his/her wishes for treatment	40 (40.0)
The timing of the call was appropriate	36 (94.7)
The duration of the call was appropriate	40 (100)
The use of the telephone for this exchange of information seemed appropriate	36 (90.0)
The exchange of information was facilitated by the fact that the caller was a physician	36 (92.3)
**Concerning the link sheet (*n*** **= 100)**	
Remembered receiving a link sheet summarizing information on the patient’s health status and his or her wishes for care	51 (51.0)
Considered the link sheet useful	39 (76.5)
**Concerning CREDO exchanges in general (*n*** **= 33 to 85)**	
**Satisfaction (“satisfied” or “rather satisfied”) with CREDO exchanges (*n*** **= 58)**	49 (84.5)
**The exchanges, during the telephone call and via the link form, provided information concerning the patient (*n*** **= 75)**	38 (50.7)
**Classification of information areas where CREDO exchanges have brought new data (*n*** **= 38)** ^a^	
Possible or expected side effects of specific treatments	25 (65.8)
Future specific therapeutic management (chemotherapy, radiotherapy)	20 (52.6)
Treatments maintained after hospital discharge	19 (50.0)
Possible or expected complications	19 (50.0)
What to do if complications and/or adverse effects occur	19 (50.0)
General state at discharge (WHO score)	16 (42.1)
Precise carcinological situation (type of cancer, location of metastases)	16 (42.1)
Patient’s wishes regarding the place of care in case of complications	15 (39.5)
Dates of the next scheduled consultations and/or hospitalizations	12 (31.6)
Reason for current hospitalization	9 (23.7)
Concomitant pathologies	6 (15.8)

^a^ multiple choice question

### Information about the link form

Half of the GPs_ref_ (51%) remembered receiving a link form summarizing information on their patient’s health status and their wishes for care. GPs_ref_ who recalled this sheet considered it to be useful in 77% of the cases.

### Satisfaction about the overall contribution of CREDO exchanges

Of the 58 GPs_ref_ who commented on their satisfaction with the return home consultation, the majority (84%) were satisfied (43%) or rather satisfied (41%) by CREDO exchanges. No statistically significant association was found between GPs_ref_ characteristics and satisfaction (Table 1).

In total, 75 GPs_ref_ answered the question on the contribution of CREDO exchanges in terms of new information (Table 2). Among them, 51% actually learned information about their patient’s state of health and/or their care preferences.

When the CREDO exchanges made it possible to transmit new information to the patient’s GP_ref_ (*n* = 38) (Table 2**),** it was most often information about the adverse effects of specific treatments (66%) or about possible or expected complications (*n* = 50%), and what action to take if necessary (*n* = 50%); about future specific care (*n* = 53%) and about treatments maintained after hospital discharge (*n* = 50%).

In the multiple-choice question on their perceived value(s) of direct systematic exchanges between patients, GP_ref_ and GP_onc_ (*n* = 85) (Table 3), GPs_ref_ most often emphasized the updating of patient information (82%), the rapidity of information transmission (81%) and the interest in anticipating emergency situations (69%).

**Table 3:** Details of the responses to the questionnaire: GP_ref_ satisfaction with direct exchanges before the patient returned home and aspects of the practice impacted after the patient returned home

	N (%)
**Responded to the survey in general (*n*** **= 100)**	
**Concerning CREDO exchanges in general (*n*** **= 33 to 85)**	
**Perceived interests of a systematic direct exchange between patient, referring physician and hospital physician, before the patient returned home (*n*** **= 85)** ^a^	
Systematic updating of information	70 (82.4)
Speed of information transmission	69 (81.2)
Anticipation of emergency situations	59 (69.4)
Information grouping, via the personalized link sheet	51 (60.0)
Reconciliation between the referring physician and the hospital team	49 (57.6)
Easy access to information, via the link sheet	47 (55.3)
Humanizing the relationship between the attending physician and the hospital team	46 (54.1)
Adaptation to the patient’s wishes	40 (47.1)
**Aspects of the practice impacted by the exchanges after the patient returned home (*n*** **= 33)** ^a^	
Management of side effects and/or complications	22 (66.7)
Patient Communication	19 (57.7)
Management of the patient’s symptoms	16 (48.5)
Involvement in patient management	15 (45.5)
Relationship with the hospital’s health care team	11 (33.3)
Communication with the patients’ family and friends	10 (30.3)
Relationship with the home care team	10 (30.3)
**Perspectives (*n*** **= 93)**	
Would request such exchanges for any cancer patient, metastatic or not	87 (93.6)
Would request multiple exchanges of this type for the same patient, should medically significant events occur	83 (90.2)
This type of initiative can improve the general practice coordination	85 (94.4)

^a^ multiple choice question

Among the 90 GPs_ref_ who commented on the potential impact of the CREDO initiative on the general practice-hospital coordination (Table 3), 94% thought that it could improve this coordination.

### Perspectives

When asked whether they would be willing to participate in such a consultation for any patient with cancer, whether metastatic or not (Table 3), 94% of the GPs_ref_ were in favour of this experimental consultation. Concerning their willingness to participate in such consultations on several occasions during the treatment of the same patient, in the event of the occurrence of medically significant events, 90% of the GPs_ref_ were in favour.

Fifty-seven GPs_ref_ responded the last open-ended question of the study.

## Discussion

### Main results

The return home consultation experimented in the CREDO trial generated good satisfaction among GPs_ref_. Among those who were dissatisfied, a majority of GPs_ref_ had actually forgotten about the intervention. The practical modalities of the CREDO exchanges, a telephone call and a link form, were both reported as having good feasibility. The evaluation of the benefits of CREDO exchanges is aimed at improving general practice-hospital care coordination. Overall, the feasibility of the return home consultation as a whole was reported as good and as having positive expected practical consequences.

### Strengths and weaknesses

The first strength is the originality of this work. To our knowledge, this is the first study on a general practice - hospital care coordination system made for and by GPs. Moreover, the choice to conduct the questionnaire preferably by telephone, followed by various reminders (by e-mail and post) resulted in a good overall response rate (55%), even if response rate was highly variable and around 30% for some questions. It is very difficult to evaluate the response rate of GPs to a telephone questionnaire because the data seem to vary widely in the literature. Our questionnaire was validated by physicians with methodological expertise in epidemiology and already involved in improving care coordination in oncology.

Finally, the researcher conducting the telephone interviews was external to the CREDO study research team, which may have reduced the subjectivity bias inherent in a study assessing satisfaction.

Our first limitation is that our sample of responding GPs is not representative of French GPs. Indeed, the population of French GPs was 52.41% male as of 1 January 2019 (67% in our study), and the average age was 51.2 years (49.94 years in our study) [17]. The number of university lecturers in France was 10,736, or 10.5% of GPs (28% in our study). Young age and participation in student training are two factors known to favour participation in research [18, 19]. This may have increased the participation of GPs in the CREDO scheme and constituted a selection bias in our study. Further work would therefore be necessary to be able to generalise the results of our study to the whole population of French GPs.

Our study required the GPs to make an effort to remember an event that occurred 6 to 22 months previously, generating a memory bias. Half of the participants were not able to remember receiving the link form, suggesting that the timing of survey implementation should have been closer to the CREDO study.

As the response rate seems to be inversely proportional to the time needed to complete it, the length of our questionnaire may have been another weakness of our work.

Finally, our study may have suffered from a desirability bias among respondents, i.e. the expression of a favourable opinion linked to the desire to present oneself as a competent professional to the researcher who conducted the interviews.

### About the phone call

Shen et al. showed in 2015 that communication between GPs and oncologists could be improved, particularly just after the diagnostic stage [20]. In 2017, the work of Dossett et al. again highlighted the shortcomings of general practice - hospital communication and the fact that direct contact with oncologists (via a personal telephone number or e-mail) was associated with improved communication and satisfaction among GPs [21]. The telephone tool was appreciated by the GPs in our study, allowing direct and rapid contact for the exchange of information with the oncologist.

The use of a GP_onc_ as the contact person during the consultation was considered a factor favouring the exchange of information. There are few studies in the literature concerning the role of a physician as the main interlocutor in general practice - hospital coordination. To our knowledge, only the Canadian study by Sisler et al. dating from 2009 studied this parameter [16]. One of the interventions in their program consisted in the assignment of a “lead physician”, with additional training in oncology, to each primary care clinic. His/her role was to foster the link with the referral center, pass on information to other health professionals and assist in oncology management. Their results were very encouraging, with 69% of GPs seeing an improvement in care coordination and 56% of GPs perceiving a benefit from the presence of the “lead physician”. This “lead physician” and the GP_onc_ defined in our study, are not strictly comparable. While the “lead physician” is stationed in a primary care center and becomes the local resource person for oncology, the GP_onc_ in the CREDO study assumes his/her role as an information carrier from the hospital. In any case, both studies have shown an improvement in GP satisfaction when a physician embodies the primary care - hospital coordination.

### About the link form

The first shortcoming of traditional mail is the delay in transmission to the GP [22–26]. In addition, in traditional letters sent from the hospital to the referring GP, important information for GPs is frequently missing [26], and these letters are sometimes difficult to read, due to a lack of structure and paragraphs that are too long and too detailed [23]. The CREDO link form is designed to be filled in quickly, at the patient’s bedside, and sent as soon as possible after hospital discharge. It has been designed with standardized information for all patients. To our knowledge, no study on GPs views of such a form has been published. The literature is abundant on the usefulness of sending GPs information sheets on the side effects of anti-neoplastic treatments [14, 27, 28]. In order to ensure that GPs were informed on this point, this type of sheet was systematically attached to the CREDO link form.

### Perspectives

To the best of our knowledge, this project is the first study of a care coordination system between general practice and the hospital carried out for and by general practitioners in the management of patients with cancer. However, other models of coordination involving GPs and hospital care have been studied in cancer and other chronic conditions. A few examples enrich our thinking and highlight the need for multidisciplinary teams to optimize the patient’s care pathway.

In cancer, Grunfeld et al. [29] conducted an RCT of 296 women with breast cancer to assess the effect on patient satisfaction of transferring the primary responsibility for follow-up of women with breast cancer in remission from hospital outpatient departments to general practice. Breast cancer patients were more satisfied with follow-up in general practice than in hospital outpatient departments. This study shows that patients have a better experience of their care, particularly at the psycho-social level, when good communication is provided by health professionals. They want to be more informed about their disease and treatments and to be more involved in the decision-making process. These results underline the fact that quality information transmitted from specialised oncology care centres to the GP can contribute to better support for patients in their care and make them more proactive in their management.

In other chronic conditions such as stroke, the systematic review by Mitchell et al. [30] included 18 papers and aimed to assess the impact of coordinated multidisciplinary care in primary care, represented by the provision of formal care planning by primary care teams or shared between primary and secondary teams, compared with usual care for stroke patients. Multidisciplinary care planning does not appear to clearly improve the care of stroke patients, but it may have benefits, particularly in terms of the division of tasks between primary and secondary care teams. Further studies on the impact of GPs’ active involvement in multidisciplinary care planning seem necessary as their role was poorly described in the literature reviewed. By collecting GPs’ satisfaction with the CREDO experimentation, our study allows us to collect the coordination modalities desired by GPs and could thus contribute to better involving them in the patients’ care pathway.

Finally, if we extend the study to the context of palliative care, two studies provide food for thought. Quill and Abernethy [31] described a model of coordinated palliative care in which the primary care physician could manage many palliative care problems, initiating a palliative care consultation for more complex or refractory problems. By promoting communication and skill sharing between primary care and specialist palliative care teams, this model would allow easier access to specialist palliative care to address problems of physician demographics and increased patient need due to longer life expectancy and increased prevalence of chronic conditions.

A systematic review by Carmont et al. [32] included 17 articles to assess the effectiveness of interventions designed to engage GPs and secondary specialist services in integrated palliative care. They showed that sharing care between GPs and specialist palliative care teams can reduce hospital admissions and maintain patients’ functional status. However, the effectiveness of integrated palliative care models remains to be evaluated.

In the light of our results and the reflections in the literature on the topic of care coordination involving the GP, certain developments of the experimental CREDO system should be considered. For example, the telephone call could be scheduled, by agreeing a telephone appointment with the GP. The information from the liaison sheet could be included in the hospital discharge letters. At present, GP_onc_ work in specialist cancer centres and often have a second activity in primary care. This gives them a comprehensive view of the cancer patient’s care pathway. In addition, the fact that they are trained in oncology makes them well suited to answering the questions of the GPs_ref_ concerning diagnosis, prognosis, the various treatments and supportive care. However, there are not enough GP_onc_ and their mission is broader than just coordinating care for patients hospitalised in the active phase of treatment. As the CREDO trial is financed by the French Ministry of Health, we hope that the results will enable a reflection about the effective and permanent establishment of a physician dedicated to coordination between general practice and hospital.

## Conclusion

Our study assessed the satisfaction of referring GPs with a system of direct coordination between hospital and general practice for patients with metastatic cancer. The CREDO trial is experimenting with a direct way of transmitting information between physicians. Our study has shown that this strategy is feasible and well received by GPs. However, it can only be carried out by the referring care centre and by a sufficiently trained professional.

## Supplementary Information

**Additional file 1.**

## Data Availability

The data supporting the conclusions of this study will be available from the Clinical Trials Unit of the Institut Universitaire du Cancer de Toulouse (IUCT) but there are restrictions on the availability of these data, which will therefore not be publicly available. The data will be, however, available from the corresponding author upon reasonable request and with the permission of the IUCT.

## Abbreviations

GP: General practitioner
GP_ref_: Referring general practitioner.
GP_onc_: General practitioner with specific skills in oncology
