# Supplementary material for: Experimental system of care coordination for the home return of patients with metastatic cancer: a survey of general practitioners
Source: BMC Prim Care. 2022 Nov 17;23:283. doi: 10.1186/s12875-022-01891-9 (PMC9673376; doi:10.1186/s12875-022-01891-9)
Supplement: Supplementary file 1 — Additional file 1. [file 12875_2022_1891_MOESM1_ESM.docx]

**Questionnaire to collect the opinions of general practitioners**

Opinion of general practitioners on an experimental Return Home Concertation (CREDO) for patients with metastatic cancer

1. Do you remember receiving a call [from the Oncopôle/ Hospital of Auch] in the context of this study, to exchange information on your patient's state of health and his/her wishes for treatment?

○ Yes

○ No

If not, go directly to question 6.

2. Was the timing of the call appropriate?

○ Yes, I was not bothered by the call

○ Yes, although I would have preferred another time

○ No, the call should have been made at another time.

○ No, the call really bothered me while I was working.

3. How long was the call?

○ Too long

○ Appropriate

○ Too short

4. Does the use of the telephone for this exchange of information seem relevant to you?

○ Yes

○ No

For what reason(s)?

5. Was the exchange of information facilitated by the fact that the caller was a physician?

○ Yes

○ No

For what reason(s)?

6. Do you remember receiving a link sheet summarizing information on your patient's health status and his or her wishes for care?

○ Yes

○ No

If not, go directly to question 8

7. Do you consider this link sheet useful?

○ Yes

○ No

For what reason(s)?

8. Did these exchanges, during the telephone call and via the link form, provide you with any information concerning your patient(s)?

○ Yes

○ No

If so, which ones?

Several possible answers.

○ General state at discharge (WHO score)

○ Precise carcinological situation (type of cancer, location of metastases)

○ Reason for current hospitalization

○ Future specific therapeutic management (chemotherapy, radiotherapy)

○ Concomitant pathologies

○ Treatments maintained at hospital discharge

○ Possible or expected complications

○ Possible or expected side effects of specific treatments

○ What to do if complications and/or adverse effects occur

○ Patient's wishes regarding the place of care in case of complications

○ Dates of the next scheduled consultations and/or hospitalizations

○ Other

9. For you and your practice, after the patient's return home, these exchanges have been?

○ Satisfactory

○ Quite satisfactory

○ Rather unsatisfactory

○ Unsatisfactory

10. What aspects of your practice were impacted by these exchanges, after the patient returned home?

○ Management of the patient's symptoms

○ Management of side effects and/or complications

○ Patient Communication

○ Communication with the patient's family and friends

○ Relationship with the home care team

○ Relationship with the hospital's health care team

○ Involvement in patient management

○ Other

○ None

11. What are, for you, the interests of a systematic direct exchange between patient, attending physician and hospital physician, before the patient returns home?

○ Systematic updating of information

○ Speed of information transmission

○ Easy access to information, via the link sheet

○ Information grouping, via the personalized link sheet

○ Adaptation to the patient's wishes

○ Anticipation of emergency situations

○ Reconciliation between the attending physician and the hospital team

○ Humanizing the relationship between the attending physician and the hospital team

○ Other

12. Would you request such exchanges for any cancer patient, metastatic or not?

○ Yes

○ No

13. Would you request multiple exchanges of this type for the same patient, should medically significant events occur?

○ Yes

○ No

14. For you, can this type of initiative improve the general practice coordination?

○ Yes

○ No

15. Do you have any suggestions for improvements to the general practice coordination?

The following questions deal with socio-demographic data concerning you, which are essential for the statistical analysis of the results. They are strictly confidential.

1. Gender

○ Woman

○ Man

2. Age

3. Where is your practice located?

○ Urban

○ Semi-rural

○ Rural

4. How do you practice?

○ Sole practitioner

○ In a group practice of several GPs

○ In a group practice of several medical or paramedical specialties

○ In a Multidisciplinary Healthcare Home

○ Other

5. Are you a university lecturer?

○ Yes

○ No

6. In what year did you establish your practice?

7. Do you have a degree or additional training in oncology?

○ Yes

○ No

If so, which one?
